# Differential responses of avian and mammalian predators to phenotypic variation in Australian Brood Frogs

**DOI:** 10.1371/journal.pone.0195446

**Published:** 2018-04-05

**Authors:** J. P. Lawrence, Michael Mahony, Brice P. Noonan

**Affiliations:** 1 University of Mississippi, Department of Biology, University, MS, United States of America; 2 University of Newcastle, School of Environmental and Life Sciences, Callaghan, NSW, Australia; University of New England, Australia, AUSTRALIA

## Abstract

Anti-predator signaling is highly variable with numerous examples of species employing cryptic coloration to avoid detection or conspicuous coloration (often coupled with a secondary defense) to ensure detection and recollection. While the ends of this spectrum are clear in their function, how species use intermediate signals is less clear. Australian Brood Frogs (*Pseudophryne*) display conspicuous coloration on both their dorsum and venter. Coupled with the alkaloid toxins these frogs possess, this coloration may be aposematic, providing a protective warning signal to predators. We assessed predation rates of known and novel color patterns and found no difference for avian or mammalian predators. However, when *Pseudophryne* dorsal phenotypes were collectively compared to the high-contrast ventral phenotype of this genus, we found birds, but not mammals, attacked dorsal phenotypes significantly less frequently than the ventral phenotype. This study, importantly, shows a differential predator response to ventral coloration in this genus which has implications for the evolution of conspicuous signaling in *Pseudophryne*.

## Introduction

Phenotypic coloration and pattern in prey species often serves to evade or deter potential predators. These signals range from cryptic (i.e., background matching; [1]) to conspicuous (i.e., aposematism; [2]) with some signals serving both functions [3,4]. In some cases, how prey species utilize a phenotypic signal is easy to discern (i.e., cryptic coloration of many moths or conspicuous coloration of poison frogs), but in many cases, how signals are used is not clear [5]. Signal interpretation is dependent upon the observer and environmental conditions in which the signal is received. For example, there is experimental evidence that coloration in the Strawberry Poison Frog (*Oophaga pumilio*) is aposematic [6], however, when viewed by conspecifics, these colors can affect mate choice [7]. Even aposematism may be observer-specific as modeling has demonstrated that conspicuous signals to avian predators may not be conspicuous to other predators (i.e., crabs and snakes [8]). The duality of these signals can result in phenotypes that are complex, and perhaps not readily apparent as to how species use signals.

**Fig 1:**
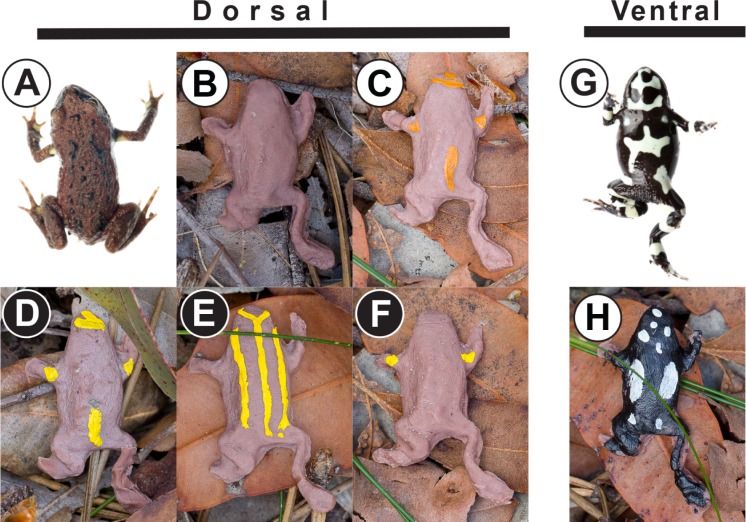
Dorsal and ventral phenotypes represented in the models. Dorsal (A) and ventral (G) phenotypes of *Pseudophryne coriacea* found in Watagans National Park. Dorsal phenotypes brown (B), orange head (C), yellow head (D), corroboree (E), and flash marks (F) represent dorsal phenotypes found in *P*. *bibronii*, *P*. *australis*, *P*. *dendyi*, *P*. *corroboree*, and *P*. *bibronii/P*. *dendyi*, respectively. The ventral reticulated (H) phenotype is found throughout the genus. Local and novel (for the study site) phenotypes are denoted by white circles with black letters and black circles with white letters, respectively.

*Pseudophryne* are small, terrestrial Australian frogs exhibiting dorsal coloration ranging from solid brown to brilliant yellow stripes on a black background (e.g., Fig 1A). As these frogs possess distasteful alkaloid toxins [9,10], this coloration is thought to be aposematic [11]. However, some *Pseudophryne* species have little conspicuous coloration, often confined to inguinal flash marks. Research on Neotropical poison frogs (Dendrobatidae) suggests there may be an inverse relationship between toxicity and conspicuousness [12,13], and it is certainly possible that this may be true of *Pseudophryne*.

Further complicating matters, however, is that all *Pseudophryne* have conspicuous black-and-white ventral patterns (Fig 1G). This coloration has also been hypothesized to function in an aposematic manner [11] as disturbed frogs are likely to freeze position and not right themselves if flipped upside down (pers. obs.). Unlike the Neotropics [6,14], mammals (e.g., marsupial predators such as quolls, possums, and *Antechinus*) may be important predators of these frogs that contribute to signal evolution. While dorsal yellows, reds, and oranges would be effective signals for birds which are sensitive to long wavelength colors [15,16], many Australian mammals have limited ability to perceive color [17], so high-contrast signals would likely be more effective at deterring predation. Alternatively, this black-and-white coloration could be disruptive, breaking up the shape of the frog [18]. We examined how predators in eastern Australia react to natural dorsal and ventral phenotypes both known and novel to the region.

## Material and methods

This study was conducted in the Watagans National Park (33°03’S, 151°20’E) in New South Wales in July 2015. This site was chosen because three *Pseudophryne* species have been recorded from the site (*P*. *australis*, *P*. *bibronii*, and *P*. *coriacea*). We constructed 1174 replica frogs using Monster Clay® [14,19] which retains evidence of predation attempts, a method used in predation experiments of snakes [20,21], frogs [14,22], and even mice [23]. We poured melted clay into silicone molds made from plastic model replicas [24] that were 3D printed from a museum specimen of *P*. *bibronii*. Models were approximately 30mm in length (snout-to-vent). Body shape is largely conserved among *Pseudophryne* species, thus the use of *P*. *bibronii* is appropriate (see S1 Fig). All models were brown with one of six phenotypes painted on them with acrylic paint [22]. Brown *Pseudophryne* are highly variable in the shade of the dorsum, and thus we could not include that variability in our clay (see S1 Fig). For that reason, we used a single brown base color for all models. Models were constructed to represent naturally occurring dorsal patterns (though some were novel for the study site): brown, orange head, yellow head, corroboree, flash marks (Fig 1B–1F), and the reticulated ventral pattern found in all species (Fig 1H). Notably, the corroboree pattern was meant to mimic the pattern found in *P*. *corroboree* [25], a species found ~360km away, representing a novel phenotype and not meant to be an exact representation of *P*. *corroboree*, whose base color is normally black.

Models were placed on four transects separated by at least 100m (750m to 2.1km long). The six model types were randomized with one model every 3m with equal numbers of each phenotype on each transect [14,26,27]. Models were left for one week [14,28], after which models were collected, and predation attempts recorded by noting bite marks. Avian attacks were recognized by a U or V shape beak impressions as well as holes indicative of stabbing-type attacks. Mammals were identified by heterodont tooth impressions. All research was conducted under University of Newcastle Animal Care and Ethics Committee (ACEC) protocol number A-2015-513. Research in Watagans National Park was conducted under New South Wales Scientific Permit SL100190.

### Data analysis

Multiple attacks on a single models were scored as a single predation attempt as we were not able to determine whether one predator attacked multiple times or multiple predators attacked once [14]. Consecutive attacks on the same model type would be counted as one attack to avoid nonindependence of attacks [19]. We observed four instances in which the same type of predator attacked consecutive models, but in all four instances, consecutive attacks were on different phenotypes. Consequently, attacks were counted independently as attacks on different phenotypes represented different decisions made by a predator. Since missing models cannot be determined to be the result of predation or detectability by the observer, missing models (N = 129) were excluded from the analysis.

Because the frequency of predation attempts in clay model studies are typically low [14,20], a G-Test of Independence is most appropriate for examining frequency of attacks among model types. We used a G-Test of Independence to compare predation attempts among phenotypes to determine whether there were differences among phenotypes. This will allow us to examine if there are differential attacks by phenotype compared to the null expectation of no difference among phenotypes.

## Results

From 1045 recovered models, we recorded a total of 45 attacks by birds (N = 18) and mammals (N = 27; Table 1). There was no statistical difference in number of attacks among naturally occurring dorsal phenotypes (Fig 1B–1F) for birds (G_4_ = 5.6, p = 0.23) or mammals (G_4_ = 0.99, p = 0.91).

**Table 1:** Distribution of attacks for each of the six different models for birds and mammals. Models labeled with an asterisk (*) are the local phenotypes that can be currently or historically found in Watagans National Park.

	Dorsal Signals					Ventral Signals
	Brown*	Flash Marks	Yellow Head	Corroboree	Orange Head*	Reticulated*
Birds	3	3	0	2	1	9
Mammals	5	4	6	3	5	4
Total Collected	167	176	176	176	173	177
Total Missing	29	22	22	18	21	17

**Fig 2:**
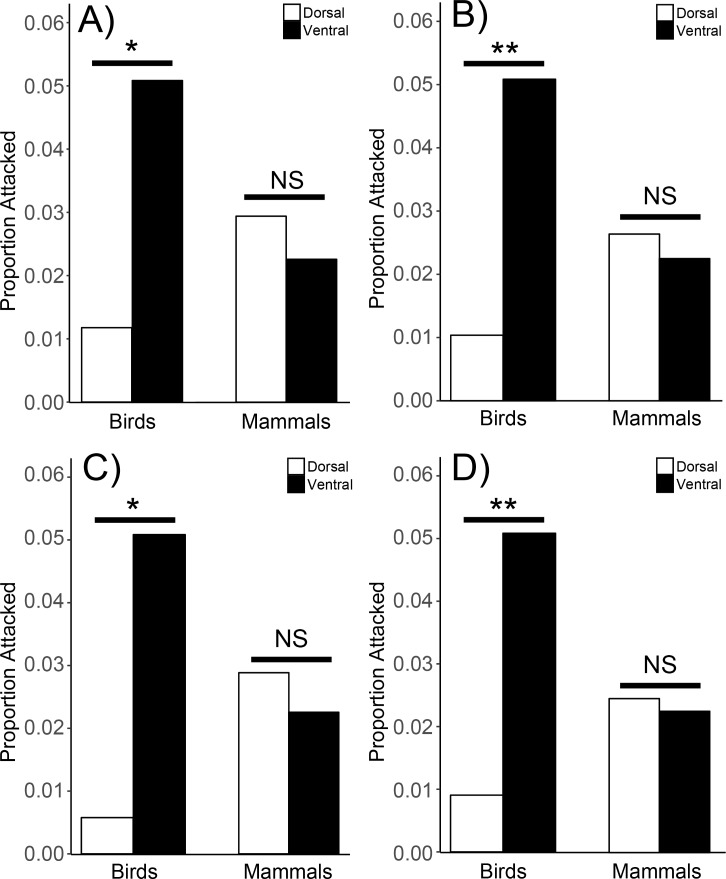
Attack proportions for birds and mammals on dorsal and ventral reticulated phenotype. When combining all local dorsal models (A), all dorsal models (B), the local conspicuous dorsal model (orange head; C), and all novel dorsal models (D). Proportions are either nonsignificant (NS) or significant (0.05 > p > 0.01, * and 0.01 > p > 0.001, **).

We further analyzed only the phenotypes that naturally occur in Watagans National Park (orange head, brown, and reticulated [ventral]) and found that dorsal phenotypes (orange head and brown) were attacked significantly less than the ventral phenotype (reticulated) by birds (G_1_ = 6.43, p = 0.01) but not mammals (G_1_ = 0.2, p = 0.65; Fig 2A). We also found birds, but not mammals, attacked the reticulated ventral phenotype significantly more when compared to all dorsal phenotypes (Fig 1B and 1C; birds: G_1_ = 9.4, p = 0.002; mammals: G_1_ = 0.18, p = 0.68; Fig 2B), the local conspicuous phenotype (Fig 1C; birds: G_1_ = 6.12, p = 0.01; mammals: G_1_ = 0.3, p = 0.59; Fig 2C), and the novel dorsal phenotypes (Fig 1D–1F; birds: G_1_ = 8.5, p = 0.004; mammals: G_1_ = 0.08, p = 0.77; Fig 2D).

Of the 1174 models placed, 129 (10.9%) were missing. Notably, the majority of these missing (71) came from a single transect which had a series of six consecutive bird attacks before most models went missing down the transect, suggesting, perhaps a bird had discovered the transect and proceeded to attack or obscure the rest of the transect. The disturbed leaf litter where missing models occurred on this transect suggests an Australian Brushturkey (*Alectura lathami*) or Superb Lyrebird (*Menura novaehollandiae*) discovered the transect and foraged along it. Excluding this transect, and we had 58 missing models of 1050 models placed (5.5%) which is consistent in clay model studies (e.g., 1.5% to 14% [6,22,27]). Nonetheless, there is no significant difference between missing models when comparing dorsal to ventral models (G_1_ = 1.24, p = 0.266; Table 1).

## Discussion

*Pseudophryne* are known for their toxins [9] and their presumed aposematic coloration [11]. While dorsal coloration can be variable within and among species, ventral coloration is far less variable. Our research suggests dorsal coloration is an important signal for avoiding avian predation. We also provide insight into the function of ventral coloration in *Pseudophryne*. While caution should be taken in interpreting these results due to the low attack rate (4.3%), this is within the range of attack rates reported in other studies. For example, of the 1218 models recovered by Hegna *et al*. (2012), a total of 91 were attacked (7.4%), but only 19 (1.5%) were avian attacks compared to 18 (1.7%) reported in this study.

We found black-and-white ventral reticulation to be ineffective in deterring avian predators. This high-contrast signal had been thought to function as a signal to mammalian predators as many Australian mammals have limited ability to discern color [17,29]. We found no difference in the attack rates of dorsal versus black-and-white signals by mammals suggesting the possibility that both dorsal and ventral signals serve an anti-predator function for mammals, though it is impossible to differentiate equal avoidance or equal disregard for signal. For the latter, it may be that traits not captured in plasticine models (i.e., odor) contribute to the deterrence of mammalian predators [30].

Plasticine models are an excellent way to assess native predator response to known and novel phenotypes of conspicuous prey [14,22,27,31], though use of models does have drawbacks. Perhaps most notably, many predators, especially avian predators, are particularly attracted to movement [32]. Indeed, when incorporating movement into clay model studies, attack rates increase for models, although conclusions for both stationary and moving models do not differ [33]. While incorporating movement in models is an important consideration, behavior of the prey species is equally important. Unlike Neotropical poison frogs [33], *Pseudophryne* are slow-moving and have the tendency to sit motionless upon detection [34], thus stationary models are an accurate representation of prey behavior.

Clearly, dorsal signals are more effective in deterring avian predators than ventral coloration, which is consistent with the higher likelihood of avian predators viewing frogs from above. Dorsal and ventral signals are equally effective against mammalian predators, possibly a product of foraging mode that likely includes chemosensory cues and/or rooting behavior that may expose frogs’ dorsal or ventral signals (they often remain immobile on their backs when exposed). This research provides an important comparison to well-studied Neotropical systems [6,14,35], that also demonstrate avoidance of dorsal signals. While numerous dendrobatid species have elaborate and colorful ventral signals, to date, no research has examined whether these signals influence predator behavior.

The reasons for model avoidance are somewhat varied and do deserve further attention. With their conspicuous coloration and alkaloid toxins, *Pseudophryne* have been proposed to be aposematic [36]. However, other anti-predator strategies, such as disruptive coloration, could explain the decreased attack rate reported in this study [18]. While not explicitly addressed in this study, future research will focus on disentangling why avian predators attack dorsal phenotypes with reduced frequency when compared to ventral phenotypes. Here, our results suggest that dorsal coloration in *Pseudophryne* has antipredator function, possibly either through aposematism or crypsis (including disruptive coloration).

## Conclusions

The research presented here represents the first exploration of signaling in *Pseudophryne* in predator deterrence. Further research will focus on whether differential avoidance based on predator type is widespread across *Pseudophryne* species or if this is solely confined to the Watagans area in Australia. Additionally, a number of myobatrachid frogs possess a black-and-white reticulated venter (i.e., *Adelotus*, *Crinia*, *Arenophryne*), indicating broad selection for this phenotype. Studies of these species could help elucidate the function of this recurring phenotype that our study suggests may play a role in deterring mammalian, but not avian, predators.

## Supporting information

S1 Fig Different *Pseudophryne* species showing variability in dorsal coloration and conservativism of body shape. A) *P*. *semimarmorata*, B) *P*. *guentheri*, C) *P*. *dendyi*, D) *P*. *australis*, and E) our clay model.
(DOCX)
