# Supplementary material for: Differential responses of avian and mammalian predators to phenotypic variation in Australian Brood Frogs
Source: PLoS One. 2018 Apr 5;13(4):e0195446. doi: 10.1371/journal.pone.0195446 (PMC5886526; doi:10.1371/journal.pone.0195446)
Supplement: S1 Fig — A) P. semimarmorata, B) P. guentheri, C) P. dendyi, D) P. australis, and E) our clay model. (DOCX) [file pone.0195446.s001.docx]

**Supplementary Information**

**
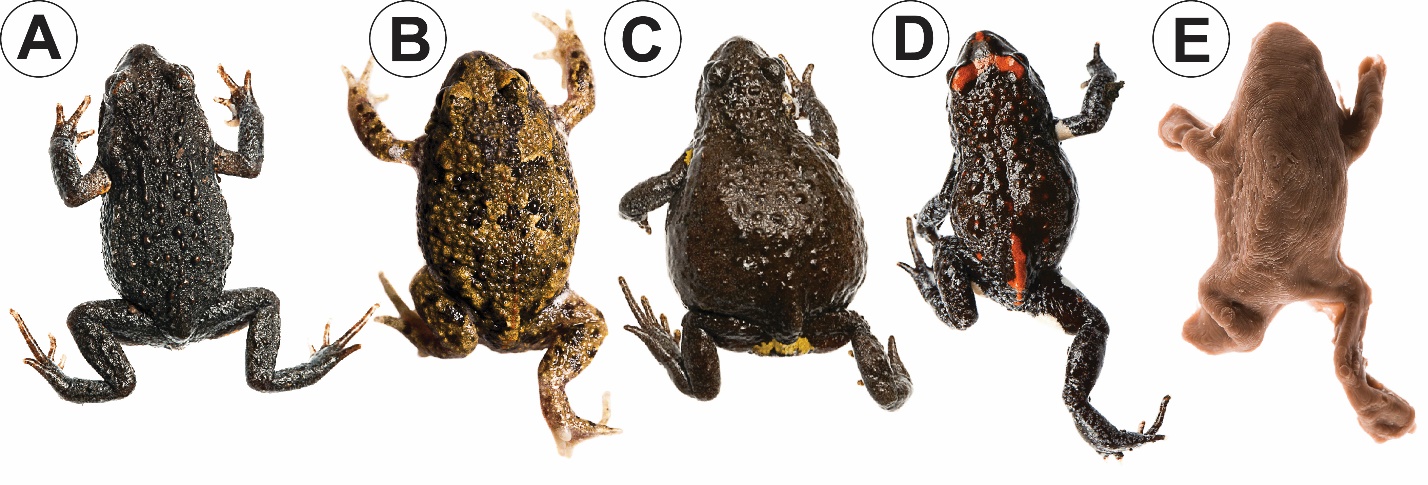
**

**Figure S1:** Different *Pseudophryne* species showing variability in dorsal coloration and conservativism of body shape. A) *P. semimarmorata*, B) *P. guentheri*, C) *P. dendyi*, D) *P. australis*, and E) our clay model.
